# Discussion of Dr. Clarke’s Paper

**Published:** 1907-02

**Authors:** C. E. Boynton


### DISCUSSION OF DR. L. B. CLARKE'S PAPER.

---

OPENED by Dr. C. E. BOYNTON.—He thought the subject a very important one and was glad Dr. Clarke had brought it up before the Society, as many of the ills seen in after life are due to improper feeding in childhood. The vitality and strong constitution of the Japanese are probably largely due to proper food when babies.

The large number of women in this country who are unable to nurse their children makes it necessary that the medical profession devote more time to this subject of infant feeding. He had made a mistake in feeding his own little son, who now has shown slight signs of rickets. Dr. Holt was the first to call attention to this matter, but was so obscure in his first effort to explain the method of modification that very few in the profession became familiar with the subject. Later this was simplified by taking the top milk. These simple rules of Dr. Clarke afford a very easy method which every man should master, as excellent results would follow their appropriate application. He deprecated the routine use of the directions given on the prepared foods, as well as some of the combinations given by physicians and old women, and wondered how any child could digest some of them.

DR. W. B. ARMSTRONG said the Society should congratulate itself upon having two of its members, Dr. Clarke and Dr. Boynton, who have devised easy methods of modifying milk for infant feeding.

DR. CYRUS STRICKLER had heard that the manufacturers were sending instructions to mothers explaining the methods of using their food or modifying milk with. This he thought was wrong.

DR. L. P. STEPHENS said that he had once thought that all modified milk was more or less of an experiment, but that this simplified the matter very greatly. As the age of an infant is not sufficient knowledge upon which to prescribe a modification, he thought the food-manufacturing companies should not send out formulæ to be used indiscriminately. He congratulated Dr. Clarke upon his paper.

DR. ARCHIBALD SMITH asked if anyone present had used sodium citrate.

DR. E. G. BALLENGER said that he had been giving it for five months to his little daughter, who is now eight months old, and felt sure she had derived much benefit from its use. The dose varies from 1 to 2 grains to each ounce of milk used. This added to the mixture just before feeding.

DR. CLARKE (closing), said he had not used sodium citrate. He showed how readily the ratio between fats and proteid could be changed by varying the depth of the milk taken for modification. The trouble in the child should be diagnosed and the ingredients given accordingly.

---

Never divide the annual ligament of the wrist. The hand is much weaker after it is divided than before.—*American Journal of Surgery.*

